# PD46 Efficacy And Safety Of Scalp Inflammation Treatment In Patients With Seborrheic Dermatitis: An Overview Of Systematic Reviews

**DOI:** 10.1017/S0266462325101797

**Published:** 2025-12-29

**Authors:** Mayra Kaizer, Thays de Andrade Apolinário, Jamilly Pereira de Almeida

## Abstract

**Introduction:**

Scalp seborrheic dermatitis (SSD) is a chronic inflammatory condition with various treatment options, including antifungal shampoos, corticosteroids, calcineurin inhibitors, and phototherapy. This study aimed to evaluate the efficacy, safety, and evidence certainty of topical and oral treatments for SSD, highlighting the importance of this research for health technology assessment (HTA) to guide healthcare decisions and optimize resource utilization.

**Methods:**

A systematic review (PROSPERO CRD42024537759) was conducted by searching PubMed, the Cochrane Library, and Embase databases through to 18 April 2024, using descriptors and terms related to SSD and study design. Systematic reviews evaluating evidence certainty (GRADE or Oxford Centre for Evidence-Based Medicine) for efficacy and safety outcomes of SSD treatments were included. Data selection and extraction were independently performed by two reviewers, and the risk of bias was assessed using the AMSTAR tool. A qualitative synthesis of the results was conducted.

**Results:**

Eight systematic reviews (80 studies on SSD) were included. Evidence quality supporting topical and oral treatments for SSD varied significantly. Ketoconazole shampoo (2 to 7%; 30 studies) and ciclopirox gel (0.77 to 1.0%; 16 studies) were the most frequently studied treatments and had moderate certainty of evidence for symptom reduction, as they were assessed in better designed studies. In contrast, other treatments commonly used in daily clinical practice were graded as having low-quality evidence (e.g., bifonazole shampoo, selenium sulfide shampoo, coal tar-ciclopirox olamine combination, gluconate ointment, succinate ointment) or very low-quality evidence according to GRADE (e.g., clobetasol propionate shampoo, zinc pyrithione shampoo, hydrocortisone).

**Conclusions:**

The review highlighted significant variability in evidence quality for SSD treatments. While ketoconazole shampoo and ciclopirox gel showed moderate evidence for symptom reduction, many commonly used treatments were supported by low or very low quality evidence. These findings underscore the importance of rigorous studies to inform HTA and support evidence-based decision-making in health care.

